# Targeting MAD2L2-dependent translesion synthesis impairs DNA damage tolerance and enhances cellular response to cisplatin

**DOI:** 10.3389/fcell.2026.1859193

**Published:** 2026-07-29

**Authors:** Philippa Jennifer Ayiku, Nomi Barda, Orly Eva Weiss, Chen Sherman, Dana Litvinov, David Fozailoff, Ido Dromi, Gadi Turgeman, Tamar Listovsky

**Affiliations:** 1 School of Biomedical Sciences, Faculty of Medicine, Ariel University, Ariel, Israel; 2 Department of Biomolecular Sciences, Weizmann Institute of Science, Rehovot, Israel

**Keywords:** cisplatin, DNA damage, MAD2L2 (REV7), small molecul inhibitor, TLS

## Abstract

Translesion synthesis (TLS) is a DNA damage tolerance pathway that enables cells to replicate across damaged DNA, thereby promoting cell survival under genotoxic stress, however, contributing to genomic instability and therapy resistance. C#3 is a small molecule that disrupts the interaction between MAD2L2 and Rev1, a key complex required for DNA polymerase ζ-mediated TLS. Here, we further investigated the impact of C#3 on MAD2L2-dependent TLS activity and cellular responses to DNA damage. Consistent with our previous findings, C#3 enhanced cellular sensitivity to cisplatin in multiple cancer cell lines and increased DNA damage signaling following treatment. Using a non-replicating plasmid assay, we demonstrate that C#3 impairs lesion bypass across DNA adducts in mammalian cells. Disruption of the MAD2L2-Rev1 axis was further supported by reduced formation of MAD2L2-Rev1 complexes, as assessed by proximity ligation assay. Functionally, cells exposed to C#3 during recovery from cisplatin treatment exhibited persistent γH2AX signaling, consistent with delayed resolution of replication-associated DNA damage. *In-vivo*, combined treatment with C#3 and cisplatin reduced tumor growth in syngeneic melanoma and triple-negative breast cancer mouse models compared with either treatment alone. Together, these findings demonstrate that pharmacological disruption of the MAD2L2-Rev1 axis impairs TLS associated DNA damage tolerance, enhances cellular responses to cisplatin-induced DNA damage, and suppresses tumor growth *in-vivo*. These results support targeting MAD2L2-dependent TLS as a potential strategy for improving the efficacy of DNA damaging chemotherapy.

## Introduction

During the past decade, significant progress has been made in immunotherapy and other targeted cancer therapies, particularly for malignancies such as melanoma, which are now often used as first-line treatments with increasing clinical success (Gaughan and Horton, 2022; Zikich et al., 2013). Nevertheless, a substantial proportion of patients still require additional chemotherapy, due to incomplete responses or disease relapse. DNA-damaging agents such as cisplatin and temozolomide (TMZ) remain widely used, especially in advanced disease settings (Bhatia et al., 2009; Cheon et al., 2020). Cytotoxic chemotherapies, including DNA-damaging alkylating agents (e.g., TMZ) and platinum analogs (e.g., cisplatin), induce intra-strand crosslinks and alkylated bases, which can lead to replication stress, DNA strand breaks, and ultimately cell death (Wang and Lippard, 2005; Jung and Lippard, 2007). However, DNA-damaging therapies can also promote mutagenesis, contributing to tumor adaptation and evolution, therapeutic resistance and increased morbidity. The mechanisms underlying intrinsic and acquired chemoresistance are diverse, involving multiple cellular pathways. Among these pathways translesion synthesis (TLS) plays a central role (Khatib et al., 2024; Cheng et al., 2024; Latancia et al., 2022).

TLS is an essential DNA damage tolerance mechanism that allows replication to proceed across DNA lesions without repairing the damage itself (Yamanaka et al., 2017; Xie et al., 2010; Chang and Cimprich, 2009). This process enables cells to adapt and survive despite the presence of DNA adducts, although at the cost of increased mutagenesis (Verma et al., 2019). The TLS machinery comprises a family of error-prone specialized DNA polymerases, including the Y-family polymerases—Rev1, Pol η, Pol ι, and Pol κ—and the B-family polymerase Pol ζ (zeta). TLS proceeds through a two-step mechanism: typically, a Y-family polymerase inserts nucleotides opposite the lesion, followed by extension of the distorted DNA by Pol ζ (Shachar et al., 2009). The Pol ζ core complex consists of the catalytic subunit Rev3 and the regulatory subunit MAD2L2 (also known as Rev7), which stabilizes Rev3 and mediates interactions with Rev1 (Sale et al., 2012; Jansen et al., 2015; Rizzo and Korzhnev, 2019). *In vivo*, TLS activity depends on MAD2L2 homodimerization and its binding to Rev1, which together ensure recruitment of the appropriate Y-family polymerase (Rizzo et al., 2018; Barda et al., 2024; Vassel et al., 2023). While lesion bypass by certain Y-family polymerases can occasionally be accurate (Avkin et al., 2006), the overall activity of Rev1 and Pol ζ is highly mutagenic, promoting chemoresistance and diminishing chemotherapy efficacy. Nevertheless, TLS confers a survival advantage to untransformed cells under genotoxic stress, as mutagenic replication is preferable to replication fork collapse and chromosomal instability (Zeman and Cimprich, 2014; Ghosal and Chen, 2013).

Given its dual role in promoting survival and mutagenesis, inhibition of the TLS pathway during chemotherapy has emerged as a promising strategy to enhance tumor sensitivity to DNA-damaging agents. Inhibition of TLS is expected to block lesion bypass, increase the accumulation of DNA damage, and trigger apoptosis. Indeed, recent studies have shown that pharmacological or genetic inhibition of TLS can sensitize cancer cells to chemotherapeutic agents and reduce the emergence of drug-resistant clones, both in cell culture and in animal models (Wojtaszek et al., 2019; Niimi et al., 2014; Vassel et al., 2020; Sakurai et al., 2020; Maggs and McVey, 2025; Kesen et al., 2025).

Here, we further characterize C#3, a small-molecule inhibitor that disrupts the MAD2L2–Rev1 interaction. Consistent with in our previous study (Pernicone et al., 2022), where we discovered and identified C#3, demonstrating the sensitization effect, disruption to the MAD2L2-Rev1 axis and direct binding of C#3 to MAD2L2, here we examine C#3’s effects on TLS activity and cellular responses to persistent DNA damage. We show that C#3 effectively sensitizes murine aggressive melanoma B16F10, murine triple-negative (TN) breast cancer 4T1 cell lines (Couto et al., 2019; Liu et al., 2024; Farhoodi et al., 2020) and mammalian melanoma A375 and colorectal carcinoma HTC116 (Koi et al., 1994) cell lines to cisplatin treatment. In addition, combined cisplatin and C#3 reduces tumor growth in melanoma and TNBC syngeneic mouse models. Building on our previous characterization of C#3, this study provides functional evidence of TLS inhibition and demonstrates therapeutic efficacy in syngeneic tumor models, providing insight into how pharmacological disruption of TLS is promising therapeutic strategy.

## Methods

### Cell lines and culture condition

B16F10, A375 and U2OS cell lines were cultured in DMEM High-Glucose medium (Diagnovum, Germany, D047-500 ML), 4T1 cells were cultured in RPMI 1640 medium (Sartorius, Israel, 01-104-1A). HCT116 cells were cultured in McCoy’s 5A (Modified) Medium (Gibco, United States, 16-600-082). All were supplemented with 10% fetal bovine serum (Diagnovum, Germany D151-500 ML), 4 mM L-glutamine (Sartorius, Israel, 03-020-1A), and 1% penicillin/streptomycin. All cell lines were maintained at 37 °C in a humidified incubator with 5% CO_2_.

### Colony survival assay

Cells were plated in 24-well plates at 5,000 cells/well (B16F10 and 4T1 lines) or 2,500 cells/well (HCT116 line). The next day, cells were treated with varying concentrations of cisplatin (APExBIO, Houston, Texas, United States, A8321) (0–10 μM), with or without 50 μM C#3 (Enamine, Kyiv, Ukraine, ZINC97017995). For the untreated control wells containing only cisplatin, the molecule was substituted with DMSO. After 48 h of cisplatin + C#3 treatment, the cells were washed 3 times with PBS, and fresh media was applied. After 4–6 days of recovery, the cells were stained with methylene blue. Plates were scanned to 8-bit TIFF images using flatbed high resolution scanner (Epson Perfection V550). Images were analyzed using ColonyArea ImageJ plugin https://imagej.net/plugins/colonyarea (Guzmán et al., 2014) using ImageJ (National Institutes of Health) area measurement tool to quantify the colony area coverage in each well. Normalization was done compared to colony area coverage in the untreated well. Each experiment was performed in triplicate and at least three independent experiments were performed for all presented concentration.

### Immunofluorescence

50,000 Cells were grown on glass coverslips in 12-well plates with the appropriate media and fixed in 4% paraformaldehyde for 10 min at room temperature. Cells were permeabilized in 0.5% Triton×100 in PBSx1 for 10 min at room temperature and then blocked in 5% BSA in 0.1% PBSx1-Tween for 1 h at room temperature. Anti-phospho-Histone H2A.X primary antibody (Merck; 05-636-25UG) diluted 1:400 in 5% BSA in 0.1% PBSx1-Tween was added for 1 h at room temperature. Fluorescent-dye conjugated secondary antibody was applied for 1 h at room temperature. The coverslips were washed between each step with PBSx1. Nuclei were stained with DAPI (1:2000 dilution) at room temperature in the dark for 3 min. Coverslips were mounted on glass slides and imaged using an Olympus 1 × 81 microscope.

### Proximity ligation assay

Protein-protein interactions were measured using the NaveniFlexTM proximity ligation assay platform (Navinci, Sweden, NF.MR.100) according to the manufacturer’s instructions. Briefly, U2OS cell lines were grown on round glass coverslips in 24-well plates and fixed in 4% paraformaldehyde for 10 min at room temperature. Cells were permeabilized in 0.5% TritonX100 in PBSx1 for 10 min, at room temperature, and then blocked with 5% BSA in 0.1% PBSx1-Tween for 1 h at 37 °C. Primary monoclonal antibodies used in the proximity ligation assay. Rev1 (mouse mAb, Santa Cruz, A-11), MAD2L2 (rabbit mAb, abclonal, A4630). The selected primary antibody pair (mouse/rabbit) was diluted 1:200 in the NaveniFlex diluent and incubated for 1 h at 37 °C. Proprietary secondary Navenibodies were applied for 1 h at 37 °C. The Navenibody oligo activation, ligation, and amplification steps were performed at 37 °C with the relevant buffers and enzyme mixes. The coverslips were washed between each step with 0.1% PBSx1-Tween. Nuclei were stained with DAPI (1:2000 dilution) at room temperature in the dark for 5 min. Coverslips were mounted on glass slides and PLA foci were imaged using the Olympus IX81 microscope. Each antibody was assessed individually using the same protocol.

### Non-replicating plasmids assay

Cells were treated with 50 µM of C#3 (or DMSO as control) for 14 h. Cells were then transfected (Lipofectamine® 2000, ThermoFisher Scientific) with a plasmids mixture containing the gap-lesion plasmid (50 ng) cisPt-GG (kanR) 5’-TCTA < GG>CCTTCT-3’ or 50 ng of BP-G (kanR) 5′-GTTCGT<G>ACGTG-3’, along with a gapped plasmid (50 ng) GP-20 (capR) without a lesion, and a carrier plasmid (1,900 ng) pID18 (ampR). Following a 10 h incubation period, plasmids were extracted (HiYield plasmid kit, Real Genomics), and used to transform *E. coli* (JM109) cells, which were then plated in parallel on kan-LB (150 µL) and cap-LB plates (150 µL), in triplicates. The ratio of kanR/capR transformants represents the extent of gap repair, which is mostly via TLS.

### Wound healing assay

To assess wound healing dynamics in A375 cells, a time-lapse wound healing assay was performed using defined 500 μm cell-free gap (Culture-Insert 2 Well in µ-Dish 35 mm, ibidi, 81176). Images were acquired every 30 min for a total of 10 h. For B16F10 cells, scratches were made using fine pipette tips and images were acquired at 0 and 10 h. All images were acquired using an Olympus X-81 light microscope. Image processing and analysis were performed using the Fiji software. Wound boundaries were manually defined using the rectangle selection tool, at 0 h, and this selection was propagated across all timepoints. The selected region was cropped from each image, converted to 8-bit grayscale, and thresholded using the Otsu method to segment the cell-free (void) region. The images were then binarized, and the Fill Holes function was applied to ensure continuity of the segmented void area. The void area was calculated from the thresholded region, using the Measure function in Fiji, and expressed as a percentage of the total selected area.

### Mouse models and treatments

C57BL/B6J and BALBc female mice were purchased from Envigo (Rehovot, Israel). Mice were housed at the Ariel University animal facility with access to food and water and libitum. At the age of 10-week-old, C57BL/B6J or BALBc mice were subcutaneously injected with two million B16F10 or 4T1 cells, respectively, into the left flank, and sequentially spread across to new housing. Once the tumors were palpable (day 1), B16F10 cells tumors were treated with direct intratumoral injection while 4T1 tumors were treated with intraperitoneal (IP) injection. Treatment was identical for both mice strains - 10 mg/kg of C#3 with or without 1 mg/kg cisplatin, 1 mg/kg cisplatin only and DMSO in saline. Mice weight was monitored to ensure no significant weight loss and tumor size was measured with digital caliper every day, if possible. Experiment ended for all groups, when tumor size reached 2.0 cm in the DMSO group. At the end of the experiment, mice were sacrificed with CO_2_, and tumors were extracted and fixed with 4% paraformaldehyde. Experimental procedures were approved by Ariel University Animal Care and Use Committee and were performed in accordance with National Institutes of Health guidelines under permissions AU-IL-2201-104-5 and AU-IL-2411-115.

### Tissue samples

Hematoxylin and Eosin (H&E) staining was done for histopathology by Dr. Loeb (Patho-Logica, Israel) on sections that were cut from tumor samples that had been fixed in 4% PFA and embedded in paraffin (4 microns). The stained tissues were examined using Olympus microscope (BX60, serial No. 7D04032) equipped with microscope’s Camera (Olympus DP73, serial No. OH05504) at objective magnifications of X1.25 and X10.

### Statistical analysis

All graphs and statistical analysis were performed using GraphPad Prism 10.5.0. For t-test, normality assessed Shapiro-wilk test.

## Results

### C#3 sensitizes cancer cell-lines cisplatin

**FIGURE 1:**
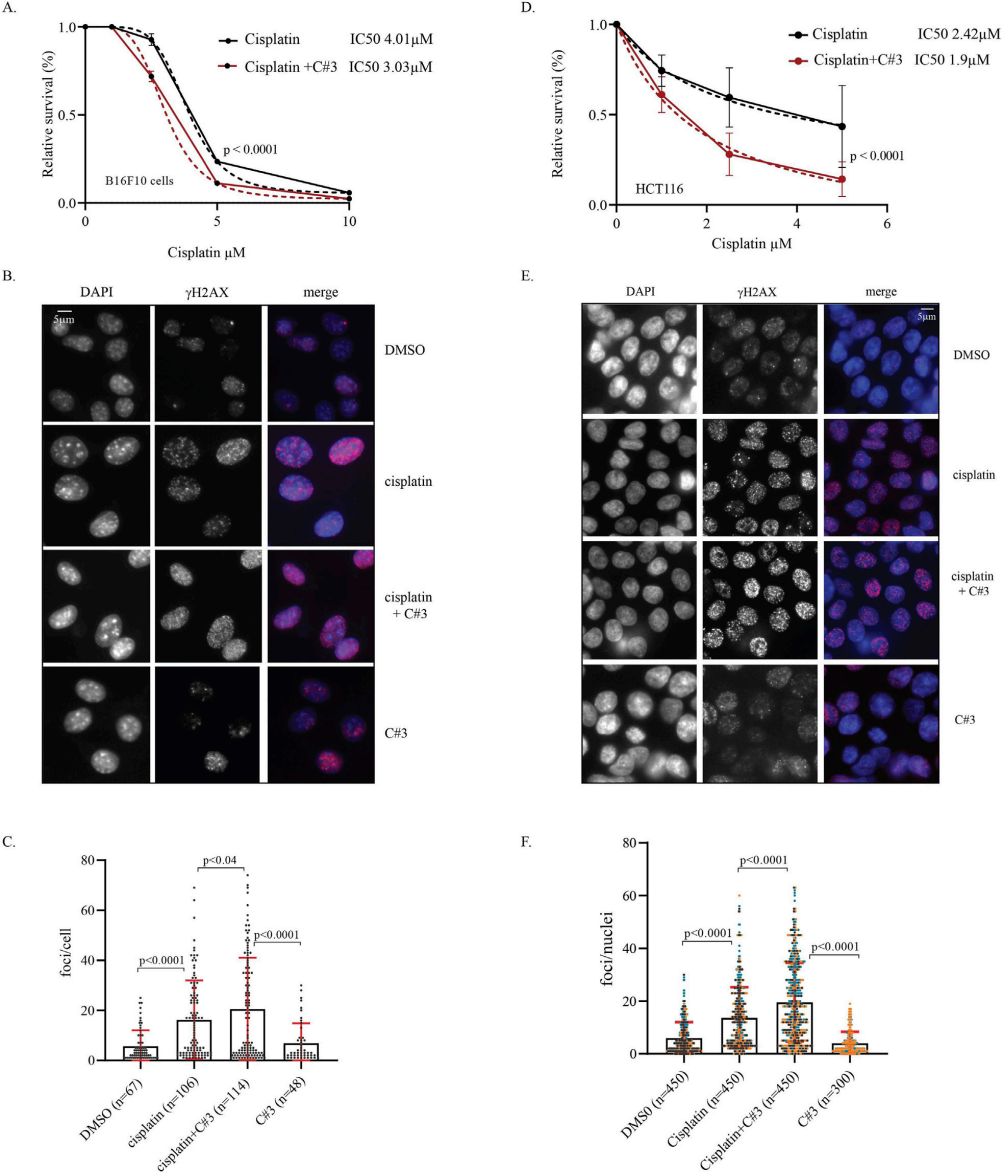
Combined cisplatin and C#3 treatment sensitize cells to cisplatin treatment and elevate DNA damage signal. **(A)** Colony survival assay of B16F10 mouse melanoma cell line presenting enhanced cell death in combined treatment. **(B)** Representative figures of γH2AX foci in B16F10 cells following 16 h treatment with cisplatin (5 μM), with or without C#3 (50 μM) at different treatments as indicated. **(C)** B16F10 cells quantification of foci/cell in each treatment. Separate data from each experiment was not available. **(D)** Colony survival assay of HCT116 human colorectal carcinoma cell line presenting enhanced cell death in the combined treatment. **(E)** Representative figures of γH2AX foci in HCT116 cells following 16 h treatment with cisplatin (5 μM), with or without C#3 (50 μM) at different treatments as indicated. **(F)** HCT116 cells quantification of foci/cell in each treatment, each experiment is presented in different colored dots. For C#3 n = 2 independent experiments. For colony survival, cells were plated in 24-well plates at 5,000 cells/well (B16F10 line) or 2,500 cells/well (HCT116 line), both cell lines were treated with 50 μM C#3 and the indicated concentration of cisplatin for 48 h; then, growth media was replaced with normal growth media with no compound. Cells were allowed to recover 4–5 days before staining. Three independent experiments were done in triplicates, error bars = 1 SD, p-value was calculated by two-way ANOVA multiple comparisons. IC50 was calculated using nonlinear regression, three parameters (dashed lines). For immunofluorescence, unless indicated differently, three independent experiments were performed for each sample and the total number of cells scored indicated by (n) in the X-axis, error bars = 1 SD, p-value was calculated by one-tailed t-test with right-tailed directionality.

To investigate whether disruption of the MAD2L2-Rev1 axis by C#3 enhances cellular sensitivity to cisplatin, we assessed cell survival in mouse melanoma (B16F10), human colorectal carcinoma (HCT116) and mouse triple-negative breast cancer (4T1) cells using colony formation assays, and in human melanoma (A375) cells using an XTT proliferation assay. For colony formation assays, cells were exposed to increasing concentrations of cisplatin (0–10 μM) in the presence or absence of a fixed concentration of C#3 (50 μM) for 48 h. Following treatment, cells were washed and allowed to form colonies for an additional 4–6 days. In all three cell lines, co-treatment with C#3 increased sensitivity to cisplatin, resulting in reduced colony formation compared with cisplatin treatment alone (Figures 1A,D; Supplementary Figure S1A). This effect was reflected by a reduction in the calculated cisplatin IC50 values (Table 1). Similarly, A375 cells exposed to identical drug combinations also exhibited increased sensitivity to cisplatin, and reduction in cisplatin IC50 values (Supplementary Figure S1B; Table 1). Across all cell lines examined, co-treatment with C#3 resulted in an approximately 20%–40% reduction in cisplatin IC50 values. Importantly, treatment with C#3 alone (50 μM) under colony formation assay conditions did not affect cell survival (Supplementary Figure S1C).

**TABLE 1:** IC50 (μM) with 95% confidence intervals, IC50 reduction and model used.

Cell line	Cisplatin IC50	95% CI	Cisplatin+ C#3 IC50	95% CI	Reduction in IC50 (%)	Model
B16F10	4.01	3.84 to 4.19	3.03	3.01 to 3.2	25%	Nonlinear regression
HCT116	2.42	0.74 to 15.82	1.9	1.16 to 3.24	22%	Nonlinear regression
4T1	8.28	5.42 to 11.15	4.95	2.63 to 7.27	41%	Nonlinear regression
A375 (XTT)	5.93	3.77 to 10.21	4.96	3.92 to 6.37	34%	Nonlinear regression

We next examined whether the enhanced sensitivity to cisplatin was associated with altered DNA damage responses, as previously reported in lung cancer cells treated with cisplatin and C#3 (Pernicone et al., 2022). γH2AX foci, a marker of DNA damage signaling and DNA double-strand break-associated responses (Bouquet et al., 2006; Mah et al., 2010) were quantified in B16F10, HCT116, and A375 cells following 16 h treatment with cisplatin (5 μM), in the presence or absence of C#3 (50 μM). In all three cell lines, co-treatment with C#3 and cisplatin resulted in significantly higher numbers of γH2AX foci compared with cisplatin treatment alone, whereas C#3 alone did not cause elevation in γH2AX levels (Figures 1B,C for B16F10; Figures 1E,F for HCT116; Supplementary Figures S1D,E for A375).

Together, these findings demonstrate that C#3 enhances the cellular response to cisplatin-induced genotoxic stress and increases cisplatin sensitivity across multiple cancer cell types, with different DNA repair capacities. The increased γH2AX signaling observed following combined treatment is consistent with enhanced DNA damage responses induced by cisplatin in the presence of C#3.

### C#3 reduces TLS complex formation and inhibits TLS in cells

Persistent γH2AX signaling is commonly used as an indicator of ongoing DNA damage responses and delayed resolution of DNA lesions. To investigate whether disruption of the MAD2L2-Rev1 axis by C#3 affects recovery from cisplatin-induced DNA damage, U2OS and HCT116 cells were treated with a low concentration of cisplatin (1 µM) for 24 h, followed by a 24 h recovery period in the presence or absence of C#3 (50 µM). DNA damage responses were assessed by quantifying γH2AX foci per nucleus and monitoring RAD51 and MAD2L2 protein levels.

**FIGURE 2:**
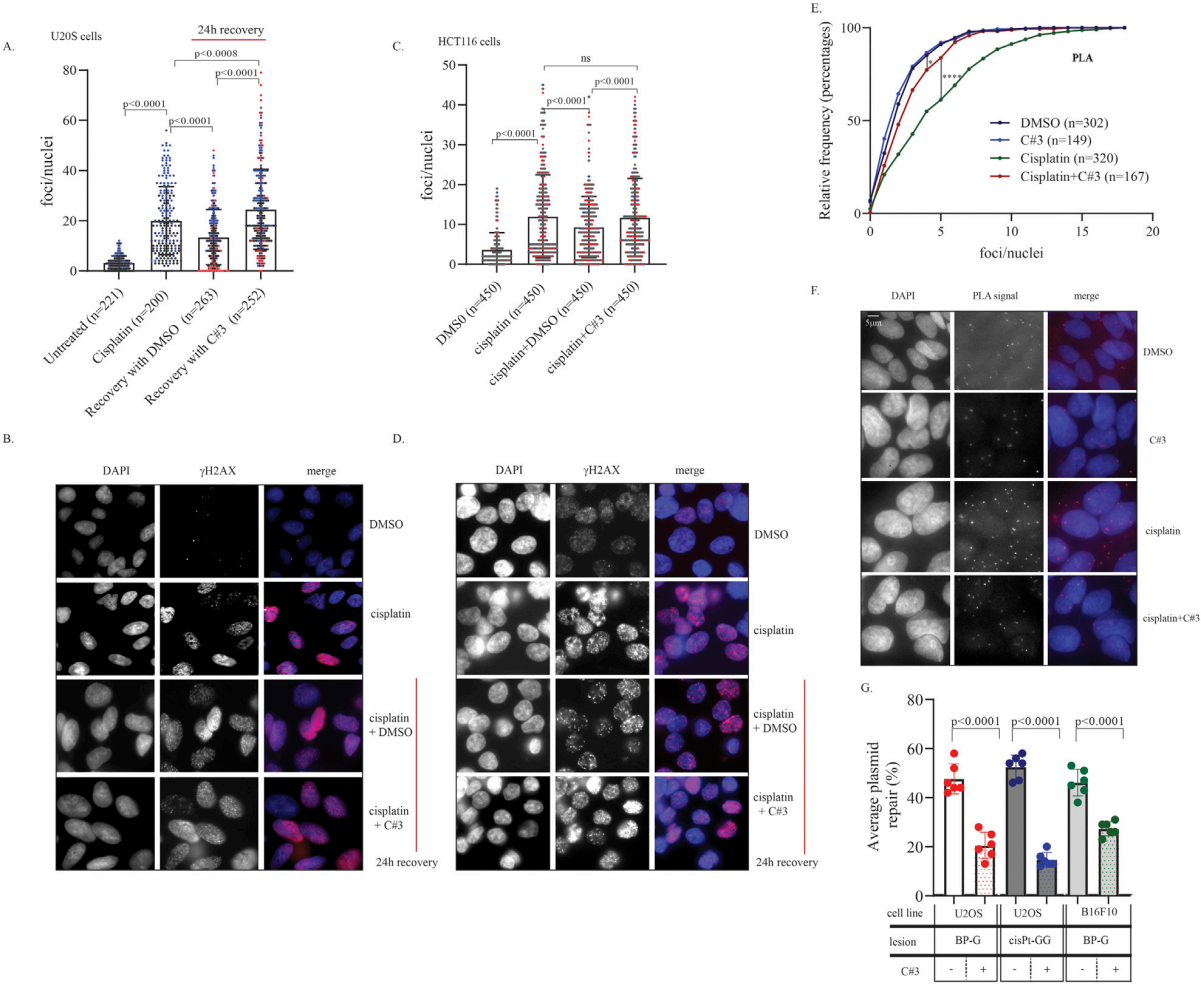
C#3 reduces DNA damage repair and inhibits TLS activity. **(A)** U2OS cells were exposed to 1 µM cisplatin for 24 h and allowed to recover 24 h in fresh medium with or with 50 μM C#3 treatment. Quantification of foci/cell in each treatment present elevation of γH2AX foci when cells were treated with C#3 after exposure to cisplatin. Each experiment is presented in different colored dots. For DMSO and cisplatin samples n = 2 and for other samples n = 3 independent experiments, total number of cells scored indicated by (n) in the X-axis, error bars = 1 SD, p-value was calculated by one-tailed t-test with right-tailed directionality. **(B)** Representative figures of γH2AX foci in U2OS cells at different treatments as indicated. **(C)** HCT116 cells were exposed to 1 µM cisplatin for 24 h and allowed to recover 24 h in fresh medium with or with 50 μM C#3 treatment. Quantification of foci/cell in each treatment present elevation of γH2AX foci when cells were treated with C#3 after exposure to cisplatin. N = 3 independent experiments. Each experiment is presented in different colored dots., total number of cells scored indicated by (n) in the X-axis, error bars = 1 SD, p-value was calculated by one-tailed t-test with right-tailed directionality. **(D)** Representative figures of γH2AX foci in HCT116 cells at different treatments as indicated. **(E)** Quantification of the PLA signal indicating MAD2L2-Rev1 complex formation. Cells that were co-treated with cisplatin (10 µM) and C#3 (100 µM), present reduced number of foci/nuclei. N = 3 independent experiments, total number of cells scored indicated by (n) in the graph legend, error bars = 1 SD, p-value was calculated by one-tailed t-test with right-tailed directionality. Same data is presented in Supplementary Figure S2D as scattered dots over bar chart, separate data from each experiment was not available. **(F)** Representative images of the PLA foci in each treatment. **(G)** Non replicating plasmid assay for assessing TLS activity across BP-G and cisPt-GG in U2OS and B16F10 cells (see also Supplementary Figure S2E and Supplementary Table S2). C#3 treatment reduced the number of DNA-damaged plasmids that were able to replicate. Data collected from two independent experiments, done in triplicates, error bars = 1 SD, p-value was calculated by one-tailed t-test with right-tailed directionality.

Cells exposed to C#3 during the recovery phase retained significantly higher levels of γH2AX compared with cells recovering in the presence of DMSO alone (Figures 2A,B for U2OS; Figures 2C,D for HCT116), indicating persistent DNA damage-associated signaling. Elevated γH2AX levels were observed in both cell lines despite their distinct DNA repair backgrounds, including the hMLH1-deficient HCT116 cells. Consistent with these findings, RAD51 and MAD2L2 protein levels remained elevated during recovery in the presence of C#3, while decreasing in control cells recovering with DMSO (Supplementary Figure S2A). Together, these observations indicate an altered cellular response to cisplatin-induced DNA damage in cells recovering in the presence of C#3.

To examine whether C#3 affects formation of the TLS-associated MAD2L2-Rev1 complex, we performed proximity ligation assays (PLA), enabling detection of endogenous protein-protein interactions *in situ*. U2OS cells were treated with cisplatin (10 µM) in the presence or absence of C#3 (100 µM) for 24 h, and nuclear MAD2L2-Rev1 PLA foci were quantified. Cells treated with DMSO or C#3 alone exhibited similarly low numbers of PLA foci, indicating minimal basal complex formation and no detectable induction of damage by C#3 alone. As expected, cisplatin treatment markedly increased the number of MAD2L2-Rev1 nuclear foci (Figures 2E,F; Supplementary Figure S2D), consistent with recruitment of TLS-associated factors following DNA damage. Notably, co-treatment with C#3 significantly reduced PLA foci to near-basal levels, supporting the reported disruption of the MAD2L2–Rev1 axis in cell (Pernicone et al., 2022). Single-antibody controls exhibited minimal background signal, and REV1 and MAD2LA protein levels remain similar during the PLA treatment, providing specificity to the PLA assay (Supplementary Figures S2B,C). Although cytoplasmic PLA signals were occasionally observed following cisplatin treatment, only nuclear foci were included in the quantitative analysis.

To directly assess the functional consequences of C#3 on TLS activity, we employed a well-established non-replicating plasmid assay that quantitatively measures lesion bypass in mammalian cells. TLS efficiency was evaluated across two structurally distinct DNA lesions: cisplatin-induced Pt-GG adducts and benzo [a]pyrene-derived BP-G adducts (Shachar et al., 2009). Consistent with inhibition of TLS-associated damage tolerance, C#3 reduced BP-G lesion bypass by approximately twofold in both U2OS and B16F10 cells (Figure 2G). In U2OS cells, bypass of Pt-GG lesions was strongly reduced, resulting in an approximately fourfold decrease in TLS efficiency (Figure 2G; Supplementary Table S2). In contrast, Pt-GG lesion bypass was not significantly affected by C#3 in B16F10 cells (Supplementary Figure S2E) suggesting cell-type-specific differences in TLS pathway utilization, lesion processing, or compensatory DNA damage tolerance mechanisms.

Together, the PLA and lesion bypass assays provide functional evidence that C#3 disrupts the MAD2L2-Rev1 axis and impairs TLS-associated lesion bypass in mammalian cells.

### C#3 reduces wound closure in melanoma cells

**FIGURE 3:**
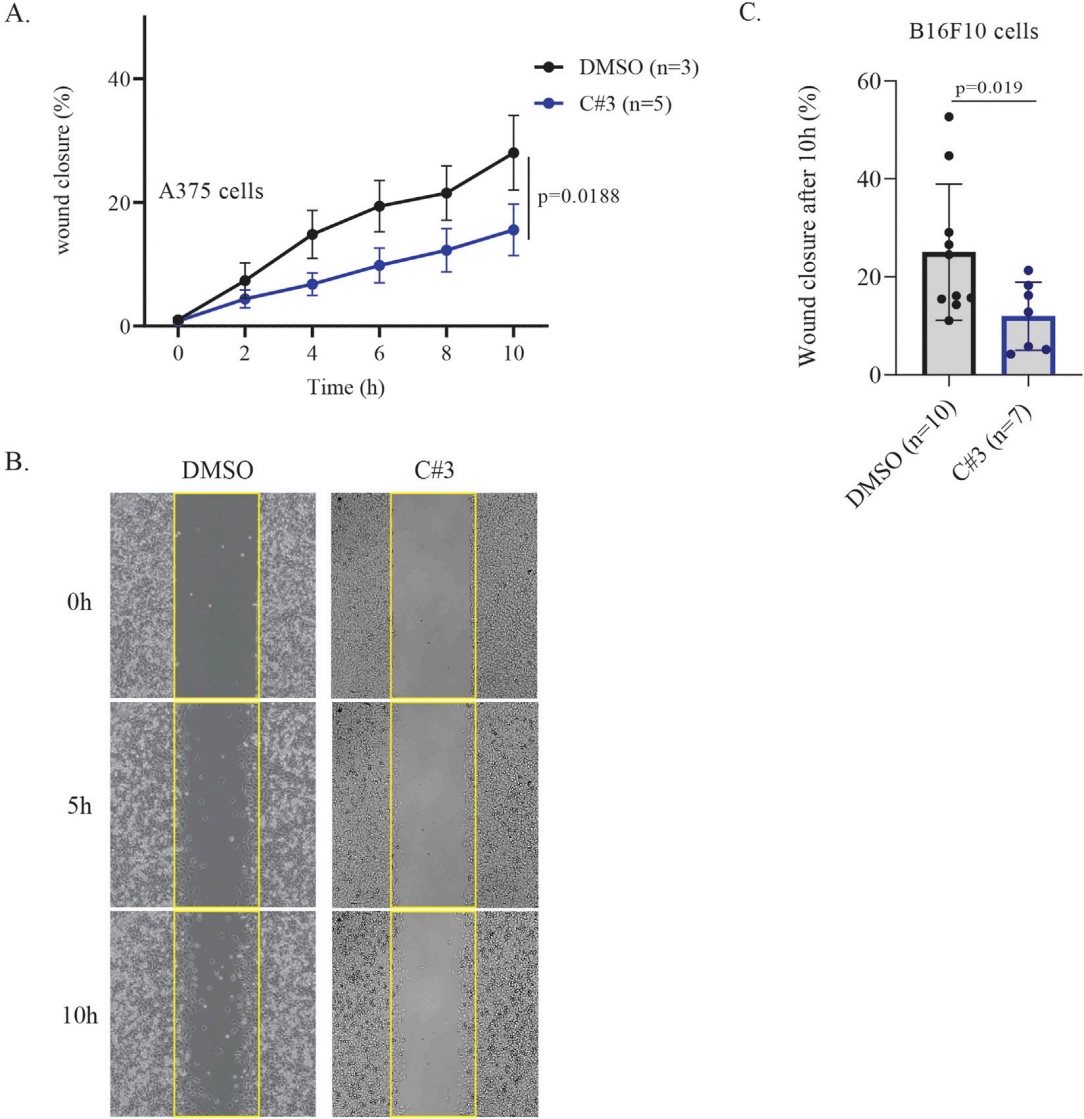
C#3 reduces cell migration. **(A)** A375 cells were seeded to confluency on defined 500 μm cell-free gap dish. Live cell imaging of the cell-free gap was performed from gap exposure (time 0 h) with or without 50 μM C#3 treatment for 10 h. The percentage of wound closure was calculated using ImagJ. **(B)** Representative frames from live cell imaging at 0, 5 and 10 h. **(C)** B16F10 cells were seeded to confluency, and scratches were made gently with a fine tip. Live cell imaging was performed with or without 50 μM C#3 treatment for 10 h. For all, the number of movies performed for each treatment is indicated by (n) in the X-axis, error bars = 1 SD, p-value was calculated from data at 10 h normalized to 0 h, using one-tailed t-test with right-tailed directionality.

MAD2L2 overexpression has been associated with cancer development, metastasis, and poor patient prognosis (Maggs and McVey, 2025; Hoshino et al., 2022; Xu et al., 2024; Zhang et al., 2019; Hayashi et al., 2025; Liu et al., 2023). Although the mechanistic relationship between MAD2L2 and metastatic behavior remains incompletely understood, several studies have reported that genetic depletion of MAD2L2 reduces melanoma cell motility and invasive potential (Hoshino et al., 2022; Liu et al., 2023; Song et al., 2021). We therefore examined whether pharmacological disruption of the MAD2L2-Rev1 axis by C#3 influences melanoma cell movement in a wound-healing assay. Wound closure was assessed in A375 and B16F10 melanoma cells using live-cell microscopy. In both cell lines, treatment with 50 μM C#3 resulted in an approximately twofold reduction in wound closure compared with control cells (Figures 3A,B for A375; Figure 3C for B16F10). Notably, C#3 alone did not significantly affect colony formation or cell viability (Supplementary Figure S1C), suggesting that the observed effect was not primarily driven by overt cytotoxicity.

While wound-healing assays may be influenced by both cell migration and proliferation, these findings indicate that inhibition of MAD2L2-dependent pathways reduces the ability of melanoma cells to repopulate the wounded area. Together with previous reports linking MAD2L2 to melanoma cell motility, these results suggest that disruption of the MAD2L2-Rev1 axis may influence cellular processes contributing to cell migration and tumor progression.

### C#3 enhances cisplatin-mediated tumor growth inhibition *in-vivo*

To evaluate the tolerability of C#3 *in-vivo*, tumor-free C57BL/6 mice were treated with vehicle (DMSO), C#3 (10 mg/kg), or a combination of cisplatin (0.5 mg/kg) and C#3 (10 mg/kg) administered intraperitoneally according to the experimental schedule. Body weight and general condition, including grooming behavior and activity, were monitored throughout the study. No significant weight differences were observed between treatment groups (Supplementary Figure S4A). At study termination, major organs, including the liver, kidneys, heart, and lungs, were collected for histopathological evaluation. No treatment-associated histopathological abnormalities were detected in C#3-treated animals (histopathological data available from Patho-Logica, Israel).

**FIGURE 4:**
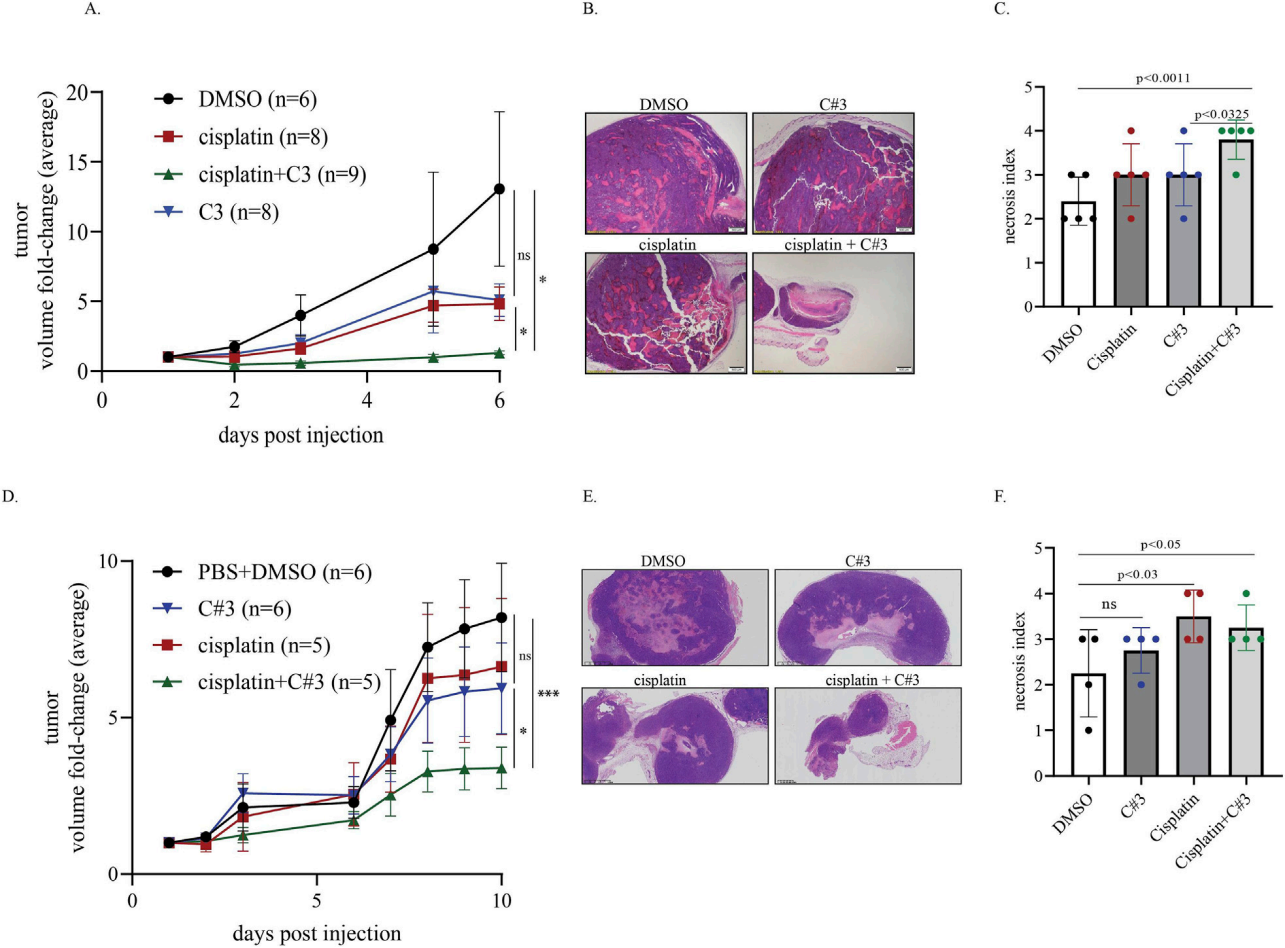
Co-treatment of C#3 with cisplatin reduced tumor growth *in-vivo*. **(A)** 2 × 10^6^ B16F10 cells were injected to the right flank of C57BL/B6J mouse and split sequentially into four treatment groups. Once tumor was palpable each group received the following treatment: DMSO contained 6 mice, cisplatin 1 mg/kg contained 8 mice, cisplatin 1 mg/kg + C#3 10 mg/kg contained 9 mice and C#3 contained 8 mice. The indicated treatment was injected into the tumor. Tumor volume fold change was calculated relative to the treatment day (day 1). Co-treatment prevented tumor growth. **(B)** H&E staining of representative tumors. Co-treated groups present large areas of necrotic tissue. **(C)** Quantification of necrotic areas found in five different tumors analyzed from all treatments. Error bars = 1 SD, p-value was calculated by two-way ANOVA. **(D)** 2 × 10^6^ 4T1 cells were injected to the right flank of BALBc mouse and split sequentially into four treatment groups. Once tumor was palpable each group received the following treatment: DMSO contained 6 mice, cisplatin 1 mg/kg contained 5 mice, cisplatin 1 mg/kg + C#3 10 mg/kg contained 5 mice and C#3 contained 6 mice. The indicated treatment was IP injected. Tumor volume fold change was calculated relative to the treatment day (day 1). **(E)** H&E staining of representative tumors. Co-treated group presents large areas of necrotic tissue. **(F)** Quantification of necrotic areas found in four different tumors analyzed. Error bars = 1 SD, p-value was calculated by two-way ANOVA.

To investigate the anti-tumor activity of C#3, two syngeneic mouse tumor models were employed. In the first model, B16F10 melanoma cells were implanted subcutaneously into C57BL/6J female mice. Once tumors became palpable, animals received a single intratumoral administration of vehicle, cisplatin (1 mg/kg), C#3 (10 mg/kg), or a combination of cisplatin and C#3. Combined treatment significantly reduced tumor growth compared with all other treatment groups, as reflected by tumor volume fold change measurements (Figure 4A). No significant differences in body weight were observed during the treatment period (Supplementary Figure S4B). Consistent with these findings, endpoint tumor weight was significantly lower in the combined treated group than in vehicle or single-agent treated animals, which was reflected in formation of smaller sized tumors (Supplementary Figures S4C,D). Tumors receiving combination treatment exhibited minimal growth during the first 3 days following treatment and only modest progression thereafter, whereas control tumors grew rapidly throughout the study. Treatment with either cisplatin or C#3 alone also reduced tumor growth, although to a lesser extent than the combination. Histopathological analysis revealed larger necrotic areas in tumors from the combination-treated group relative to all other treatment groups (Figures 4B,C).

To further evaluate the anti-tumor activity of C#3 in an independent model, 4T1 triple-negative breast cancer cells were implanted subcutaneously into BALB/c female mice. Once tumors became palpable, mice received intraperitoneal administration of vehicle, cisplatin (1 mg/kg), C#3 (10 mg/kg), or combination of cisplatin and C#3. Consistent with the melanoma model, co-treatment significantly reduced tumor growth compared with vehicle and single-agent treatment groups, as reflected by tumor volume fold change measurements (Figure 4D). No significant treatment-associated body weight loss was observed (Supplementary Figure S4E). Although tumor growth accelerated after day 6, tumors in the combined group remained smaller throughout the study (Supplementary Figure S4F). Consistently, endpoint tumor weight was significantly lower in the combined treated group than in vehicle or single-agent treated animals, which was reflected in formation of smaller sized tumors (Supplementary Figures S4H,H). Similarly to the melanoma model, treatment with either cisplatin or C#3 alone also reduced tumor growth, although to a lesser extent than the combination. Histological analysis demonstrated increased tumor necrosis in both cisplatin and combined treatment relative to controls (Figures 4E,F). Together, these findings demonstrate that C#3 enhances the anti-tumor activity of cisplatin in two independent syngeneic tumor models without evidence of overt toxicity under the conditions tested.

While the present study does not directly measure TLS activity within tumors, the observed enhancement of cisplatin efficacy is consistent with the proposed disruption of the MAD2L2-Rev1 axis and its role in cellular tolerance to DNA damage. Collectively, these *in-vivo* data demonstrate that pharmacological inhibition of TLS with C#3 potentiates cisplatin antitumor activity across distinct syngeneic tumor models.

## Discussion

Translesion synthesis (TLS) inhibition provides a useful approach to investigate how DNA damage tolerance pathways contribute to cellular adaptation to genotoxic stress. C#3 functions as a modulator of DNA damage responses through disruption of the MAD2L2-Rev1 axis, a key component of polymerase ζ-mediated TLS. Across multiple assays, treatment with C#3 altered cellular responses to cisplatin-induced DNA damage and delayed recovery following genotoxic stress. Importantly, C#3 alone did not induce detectable γH2AX accumulation, supporting its role as a non-genotoxic modulator of DNA damage response pathways. Consistent with our previous findings (Pernicone et al., 2022), C#3 sensitized multiple cancer cell lines to cisplatin, reducing cisplatin IC50 values by approximately 20%–40%. Co-treatment also increased γH2AX signaling following cisplatin exposure. While γH2AX is not specific to TLS inhibition and may reflect multiple forms of DNA damage-associated signaling, the observed increase is consistent with an altered cellular response to cisplatin-induced lesions in the presence of C#3. Together, these findings suggest that disruption of the MAD2L2-Rev1 axis compromises the ability of tumor cells to tolerate cisplatin-induced DNA damage, thereby enhancing cytotoxicity. Whether this strategy may also limit chemotherapy-induced mutagenesis and the emergence of drug resistance remains an important question for future studies.

Direct effects of C#3 on TLS-associated lesion bypass were demonstrated using a quantitative non-replicating plasmid assay. In U2OS cells, C#3 markedly reduced bypass of both cisplatin-induced Pt-GG adducts and BP-G adducts. In B16F10 cells, inhibition was more modest and lesion-dependent, suggesting potential cell-type-specific differences in TLS pathway utilization, expression of TLS polymerases, or compensatory DNA damage tolerance mechanisms. Although the molecular basis for these differences remains unclear, they highlight the complexity of TLS regulation across distinct cellular backgrounds. Importantly, despite the more limited effect on Pt-GG bypass in B16F10 cells, C#3 still enhanced cisplatin sensitivity and altered DNA damage responses, indicating that partial disruption of MAD2L2-dependent TLS can significantly influence cellular adaptation to genotoxic stress.

TLS inhibition was further supported by reduced formation of the MAD2L2-Rev1 complex, as measured by proximity ligation assays. C#3 significantly decreased cisplatin-induced MAD2L2-Rev1 foci while having little effect under basal conditions, consistent with disruption of damage-induced complex assembly. In addition, cells recovering from cisplatin exposure in the presence of C#3 exhibited persistent γH2AX signaling compared with control cells. While γH2AX persistence alone cannot distinguish between impaired TLS, replication stress, checkpoint activation, or delayed repair of DNA lesions, these findings are consistent with impaired recovery from cisplatin-induced damage. Together with the PLA and lesion bypass assays, these observations support the conclusion that C#3 disrupts the MAD2L2-Rev1 axis and modulates TLS-associated DNA damage tolerance pathways.

Beyond its role in DNA damage tolerance, MAD2L2 overexpression has been associated with tumor aggressiveness and metastatic progression. In the present study, C#3 reduced wound closure in melanoma cells. Although wound-healing assays may be influenced by both migration and proliferation, the short duration of the assay and the absence of detectable effects of C#3 on cell viability under the conditions tested suggest that reduced cellular motility contributed to the observed phenotype. These findings are consistent with previous reports demonstrating that genetic depletion of MAD2L2 impairs melanoma cell migration (Hoshino et al., 2022). Nevertheless, additional studies employing migration-specific assays will be required to fully define the contribution of MAD2L2-dependent pathways to tumor cell motility. Although the mechanistic link between MAD2L2 and metastasis remains incompletely defined, prior studies have implicated MAD2L2 in ferroptosis regulation and mTOR signaling. Given the established role of mTOR in tumor progression and metastasis (Xu et al., 2024; Zhou and Huang, 2011; Panwar et al., 2023), further investigation into the intersection between MAD2L2 function and mTOR pathway regulation is warranted.

In two independent syngeneic mice models, enhanced anti-tumor activity was accompanied by increased tumor necrosis and occurred without evidence of overt toxicity under the conditions tested. Notably, C#3 alone also produced measurable anti-tumor activity, despite exhibiting limited effects on cell viability in colony formation assays. These findings are consistent with previous reports demonstrating that MAD2L2 silencing by siRNA suppressed ovarian tumor growth to a similar extent as cisplatin, while co-treatment resulted in enhanced inhibition (Niimi et al., 2014). Given that tumor cells typically experience elevated levels of endogenous DNA damage and replication stress, TLS inhibition alone may compromise their ability to tolerate genotoxic stress, thereby contributing to tumor growth suppression Alternatively, MAD2L2-dependent functions outside of TLS, including roles in DNA repair pathway choice and Shieldin complex biology (de Krijger et al., 2021), may contribute to the observed anti-tumor effects. Additional studies will be required to distinguish between these possibilities. An important consideration when interpreting the *in-vivo* findings is the use of different administration routes in the two syngeneic tumor models. In the B16F10 melanoma model, C#3 and cisplatin were administered by intratumoral injection to maximize local drug exposure and evaluate proof-of-concept anti-tumor activity at the tumor site. In contrast, compounds were administered intraperitoneally in the 4T1 model to assess efficacy following systemic delivery. As drug distribution, bioavailability, and tumor exposure may differ substantially between intratumoral and systemic administration, direct quantitative comparison between the two models should be made with caution. Nevertheless, despite these differences in administration route and tumor type, one dose of combined treatment was effective and consistently produced greater anti-tumor activity than either agent alone. These findings suggest that the therapeutic benefit of disrupting the MAD2L2-Rev1 axis is not restricted to a single tumor model or route of administration and reinforce the translational potential of pharmacological TLS inhibition as a strategy to enhance platinum-based chemotherapy. However, future pharmacokinetic and pharmacodynamic studies using a uniform dosing strategy across models will be important to define drug exposure, optimize treatment schedules, and further evaluate the translational potential of C#3.

A further consideration relevant to the interpretation of C#3 activity is the inclusion of HCT116 cells, which are deficient in the mismatch repair (MMR) protein hMLH1. Loss of hMLH1 alters the cellular response to DNA damage, particularly to platinum-induced DNA lesions, and has been associated with changes in replication-associated repair and damage tolerance pathways (Fink et al., 1996; Papadopoulos et al., 1994). Although the present study did not employ direct genetic manipulation of MAD2L2 or Rev1, the observation that C#3 enhanced cisplatin sensitivity and promoted persistent γH2AX signaling in both hMLH1-deficient HCT116 cells and mismatch repair-proficient cell lines suggests that the biological effects of C#3 are not limited to a specific mismatch repair background. This finding supports the notion that disruption of the MAD2L2-Rev1 axis can influence cellular responses to DNA damage across genetically distinct tumor models. Nevertheless, future studies employing targeted genetic approaches, including MAD2L2 or Rev1 depletion, knockout and rescue experiments, will be important to further establish the specific contribution of this pathway to the phenotypes observed following C#3 treatment.

The interplay between MAD2L2 and BRCA1-deficient tumors presents additional complexity. MAD2L2 is a component of the Shieldin complex and influences DNA end-joining pathway choice (Liang et al., 2020; Noordermeer et al., 2018). Loss of MAD2L2 can restore homologous recombination in BRCA1-deficient contexts and promote PARP inhibitor resistance (Xu et al., 2015), whereas altered MAD2L2 expression has also been linked to PARP inhibitor sensitivity in other settings (Karakashev et al., 2020). Our findings suggest that TLS inhibition through MAD2L2 disruption may provide therapeutic benefit in TNBC independent of homologous recombination status. Furthermore, as highlighted for other small-molecule inhibitors (Yue et al., 2022) compounds with related pharmacophores may exhibit distinct binding modes and off-target activities despite targeting the same pathway. While our previous biochemical studies demonstrated direct binding of C#3 to MAD2L2 and disruption of the MAD2L2-Rev1 interaction (Pernicone et al., 2022) additional target-engagement and selectivity studies will be valuable to further define its mechanism of action and potential effects on non-TLS pathways.

In conclusion, our findings identify C#3 as a small-molecule modulator of the MAD2L2-Rev1 axis that alters cellular responses to DNA damage and impairs TLS-associated lesion bypass. Disruption of this pathway enhances cisplatin sensitivity, alters DNA damage signaling, reduces wound closure in melanoma cells, and suppresses tumor growth *in-vivo*. These results provide further insight into the contribution of MAD2L2-dependent TLS to cellular adaptation following genotoxic stress and support continued investigation of TLS-targeted therapeutic strategies.

## Data Availability

The original contributions presented in the study are included in the article/Supplementary Material, further inquiries can be directed to the corresponding author.
