# Supplementary material for: Targeting MAD2L2-dependent translesion synthesis impairs DNA damage tolerance and enhances cellular response to cisplatin
Source: Front Cell Dev Biol. 2026 Jul 29;14:1859193. doi: 10.3389/fcell.2026.1859193 (PMC13462230; doi:10.3389/fcell.2026.1859193)
Supplement: Supplementary file 1 [file DataSheet1.pdf]

Table S2 **TLS across cisPt-GG and BP-G in U2OS and B16F10 cells.**

Experiment 1

Cell line	Gap-lesion plasmid	Transformants Kan^R	Transformants Cap^R	Plasmid repair, %	Fold change (vs DMSO)
U2OS + DMSO	cisPt-GG	248	425	58±6%	3.62
U2OS + C#3		76	582	16±4%	
B16F10 + DMSO		119	339	37±7%	0.97
B16F10 + C#3		156	430	38±8%	
U2OS + DMSO	BP-G	204	482	44±3%	1.91
U2OS + C#3		96	453	23±5%	
B16F10 + DMSO		133	379	47±4%	1.62
B16F10 + C#3		169	538	29±3%	

Experiment 2

Cell line	Gap-lesion plasmid	Transformants Kan^R	Transformants Cap^R	Plasmid repair, %	Fold change (vs DMSO)
U2OS + DMSO	cisPt-GG	347	616	52±5%	3.71
U2OS + C#3		85	619	14±2%	
B16F10 + DMSO		182	438	38±3%	1.05
B16F10 + C#3		147	374	36±5%	
U2OS + DMSO	BP-G	249	542	47±4%	2.35
U2OS + C#3		145	580	20±4%	
B16F10 + DMSO		191	357	45±7%	1.73
B16F10 + C#3		131	449	26±3%	

Plasmid repair (%) is presented figure 2E. Fold chang is presented in S2C
