# Supplementary material for: Targeting MAD2L2-dependent translesion synthesis impairs DNA damage tolerance and enhances cellular response to cisplatin
Source: Front Cell Dev Biol. 2026 Jul 29;14:1859193. doi: 10.3389/fcell.2026.1859193 (PMC13462230; doi:10.3389/fcell.2026.1859193)
Supplement: Supplementary file 2 [file DataSheet2.pdf]

Supplementary methods

XTT Cell Proliferation Assay

A375 cells were plated on 96-well plates at 5,000 cells/well. On the next day, the cells were treated with varying concentrations of cisplatin (1-10µM), with or without 50µM of the C#3. After 24 hours of cisplatin + C#3 treatment, cells were washed once with PBS. XTT cell proliferation reagent was added to the content of each well according to manufacturer's protocol and incubated for 2 hours. The absorbances were read using Tecan microplate reader at 490nm.

C#3 toxicity Assay

Cells were plated in 24-well plates at 5,000 cells/well (B16F10 and 4T1 lines) or 2,500 cells/well (HCT116 line). The next day, cells were treated with 50µM of the C#3. For the untreated control, the molecule was substituted with DMSO. After 48 hours of molecule treatment, the cells were washed once with PBS, and media was replaced with fresh media for cells to recover. After 4-5 days of recovery, the cells were stained with methylene blue and analyzed as above. Each toxicity assay was performed in triplicate and at least three independent experiments were performed.

Western Blot and Antibodies

For immunoblotting, cells were lysed in extraction buffer containing 50 mM Tris-HCl pH 8, 150 mM NaCl, 20 mM EGTA, 50 mM NaF and 0.1% Triton X-100, supplemented with Merck® 1000× protease inhibitor (539134). Cells were lysed on ice for 30 min and cleared by centrifugation at 20,000× g for 30 min at 4 °C. Extracts were boiled in Laemmli buffer for 5 min. Equal amounts of protein sample (25µg) were loaded on 10% acrylamide gels and transferred to a nitrocellulose membrane (Amersham, Piscataway, NJ, USA). RAD51 (Abcam, ab133534) primary antibody was used at a 1:1000 dilution. MAD2L2 (abclonal, A4630) primary antibody was used at a 1:1000 dilution Actin (clone C4 69100, MP) was used at a 1:5000 dilution. HRP IgG light chain-specific anti-mouse secondary antibody (115-035-174; Jackson ImmunoResearch Laboratories, Inc., West Grove, PA, USA) or HRP IgG light chain-specific anti-rabbit secondary antibody (211-032-171; Jackson ImmunoResearch Laboratories, Inc.) was used at 1:10,000 dilution. Where indicated, quantitative data analysis was performed with the ImageJ/Fiji software (https://imagej.net/ij/index.html National Institute of Health, Bethesda, MD, USA).

C#3 toxicological effects *in-vivo*

To determine toxicological effects of C#3, 15 mice were randomly divided into 3 groups and each group received DMSO, 10mg/kg of C#3 or 10mg/kg of C#3+0.5mg/kg cisplatin. Treatment was administered IP for three times, every other day (day 1, day 3 and day 5) and mice were kept for four more days after last treatment. Mice weight was monitored daily. Tissues fixed in 4% buffered formalin were received by Patho-Logica, embedded in paraffin, sectioned at 4 μ thickness, stained with Hematoxylin and Eosin, and examined by light microscopy. Histopathological examination was conducted in the “blind method” (with no knowledge of the groups treatments). The liver (three lobes), kidney (L+R), spleen, heart and lung of all 15 animals were examined.

Supplementary figure legend

Figure S1. Combined cisplatin and C#3 treatment sensitize cells to cisplatin treatment and elevate DNA damage signal. (**A)** Colony survival assay of 4T1 mouse TNBC cell line, presenting enhanced cell death in the co-treated group. Three independent experiments were done in triplicates, cells were plated in 24-well plates at 5,000 cells/well, error bars =1 SD, p-value was calculated by two-way ANOVA multiple comparisons. IC50 was calculated using nonlinear regression three parameters (dashed lines). (**B**) XTT proliferation assay of A375 human melanoma cell line presenting enhanced cell death in the combined treatment. Three independent experiments were done in triplicates. Cells were plated on 96-well plates at 5,000 cells/well, error bars =1 SD, p-value was calculated by two-way ANOVA multiple comparisons. IC50 was calculated using nonlinear regression three parameters (dashed lines). (**C**) C#3 presents no toxicity on B16F10, HCT116 and 4T1 cells. Cells were plated in 24-well plates at 5,000 cells/well (B16F10 and 4T1 lines) or 2500 cells/well (HCT116 line). C#3 or DMSO were added for 48 hours and coverage was compared between after 4-5 days. Three independent experiments were done in triplicates, error bars =1 SD, p-value was calculated by two-tailed t-test, normalized to DMSO treated cells. (**D)** Representative figures of γH2AX foci in A375 cells following 16 hours treatment with cisplatin (5 μM), with or without C#3 (50 μM). (**E**) A375 cells quantification of foci/cell in each treatment. Three independent experiments were performed for each sample and the total number of cells scored indicated by (n) in the X axis, separate data from each experiment was not available. Error bars =1 SD, p-value was calculated by one-tailed t-test with right-tailed directionality.

Figure S2. C#3 reduces DNA damage repair and inhibits TLS activity. (**A**) RAD51 and MAD2L2 protein levels, in U2OS cells during cisplatin treatment and recovery in the presences or absent of C#3. (**B**) Representative images of the PLA foci for single monoclonal antibody control and quantification of the PLA signal. For both antibodies untreated cells presented less than one foci/nuclei. Total number of cells scored indicated by (n) at the x axis, error bars =1 SD, p-value was calculated by two-tailed t-test. (**C**) Rev1 and MAD2L2 protein levels in PLA condition. (**D**) Quantification of the PLA signal indicating MAD2L2-Rev1 complex formation. Cells that were co-treated with cisplatin and C#3, present reduced number of foci/nuclei. N=3 independent experiments, total number of cells scored indicated by (n) in the graph legend, error

bars =1 SD, p-value was calculated by one-tailed t-test with right-tailed directionality. Same data is presented in Figure 2E at relative frequency distribution, separate data from each experiment was not available. (**E**) Fold change of TLS across lesions with C#3 treatment. Data collected from two independent experiments, done in triplicates, error bars =1 SD, p-value was calculated by one-tailed t-test with right-tailed directionality. Experimental data that was used to generate figures 2G and S2E are presented in Table S2.

Figure S4. Co-treatment of C#3 with cisplatin reduced tumor growth *in-vivo*. (**A**) Five tumor-free mice received DMSO, C#3 10 mg/kg or combination of cisplatin 0.5 mg/kg + C#3 10 mg/kg. Drugs were administered intraperitoneal three times as indicated. Body weight and general condition were monitored throughout the treatment period, and no significant differences were observed compared to controls. Error bars =1 SD, p-value was calculated by mixed two-way ANOVA. (**B**) C57BL/6 mice body weight and general condition were monitored throughout the treatment period, and no significant differences were observed compared to controls. Error bars =1 SD, p-value was calculated by mixed two-way ANOVA. (**C**) By the end of the experiment, tumors were extracted cleaned and weighted. Combined treatment presented significantly smaller tumor mass compared to all other groups. error bars =1 SD, p-value was calculated by one-tailed t-test with right-tailed directionality. (**D**) Representative images of three tumors from each treatment group. (**E**) BALBc mice body weight and general condition were monitored throughout the treatment period, and no significant differences were observed compared to controls. Error bars =1 SD, p-value was calculated by mixed two-way ANOVA. (**F**) BALBc mice tumor volume as measured from day 1 until the end of the experiment day 10. From these data average tumor volume fold-change was calculated (Fig. 4D). (**G**) By the end of the experiment, tumors were extracted cleaned and weighted. Combined treatment presented significantly smaller tumor mass compared to all other groups. error bars =1 SD, p-value was calculated by one-tailed t-test with right-tailed directionality. (**H**) Representative images of three tumors from each treatment group. For all, number of mice in each treatment group is indicated by (n).
